# Protocol for a systematic review of prognostic models for the recurrence of venous thromboembolism (VTE) following treatment for a first unprovoked VTE

**DOI:** 10.1186/2046-4053-2-91

**Published:** 2013-10-03

**Authors:** Joie Ensor, Richard D Riley, David Moore, Susan Bayliss, Sue Jowett, David A Fitzmaurice

**Affiliations:** 1 Public Health, Epidemiology and Biostatistics, School of Health and Population Sciences, University of Birmingham, Edgbaston, Birmingham, B15 2TT, UK; 2 Health Economics, University of Birmingham, Edgbaston, Birmingham, B15 2TT, UK; 3 Primary Care Clinical Sciences, University of Birmingham, Edgbaston, Birmingham, B15 2TT, UK

**Keywords:** Meta-analysis, Prediction, Prognosis, Pulmonary embolism, Recurrence, Risk factors, Thromboembolism, Venous thrombosis

## Abstract

**Background:**

Venous thromboembolism (VTE) is a chronic disease, with fatal recurrences occurring in 5% to 9% of patients, yet it is also one of the best examples of preventable disease. Prognostic models that utilise multiple prognostic factors (demographic, clinical and laboratory patient characteristics) in combination to predict individual outcome risk may allow the identification of patients who would benefit from long-term anticoagulation therapy, and conversely those that would benefit from stopping such therapy due to a low risk of recurrence. The study will systematically review the evidence on potential prognostic models for the recurrence of VTE or adverse outcomes following the cessation of therapy, and synthesise and summarise each model’s prognostic value. The review has been registered with PROSPERO (CRD42013003494).

**Methods/design:**

Articles will be sought from the Cochrane library (CENTRAL, CDSR, DARE, HTA databases), MEDLINE and EMBASE. Trial registers will be searched for ongoing studies, and conference abstracts will be sought. Reference lists and subject experts will be utilised. No restrictions on language of publications will be applied. Studies of any design will be included if they examine, in patients ceasing therapy after at least three months’ treatment with an oral anticoagulant therapy, whether more than one factor in combination is associated with the risk of VTE recurrence or another adverse outcome. Study quality will be assessed using appropriate guidelines for prognostic models. Prognostic models will be summarised qualitatively and, if tested in multiple validation studies, their predictive performance will be summarised using a random-effects meta-analysis model to account for any between-study heterogeneity.

**Discussion:**

The results of the review will identify prognostic models for the risk of VTE recurrence or adverse outcome following cessation of therapy for a first unprovoked VTE. These will be informative for clinicians currently treating patients for a first unprovoked VTE and considering whether to stop treatment or not for particular individuals. The conclusions of the review will also inform the potential development of new prognostic models and clinical prediction rules to identify those at high or low risk of VTE recurrence or adverse outcome following a first unprovoked VTE.

## Background

Venous thrombosis is a chronic disease, and recurrent events are fatal in approximately 5% to 9% of patients [1]. Most recurrences are easily preventable using antithrombotic therapy, and it is therefore of great importance that demographic, clinical or laboratory patient characteristics associated with an increased risk of recurrence or adverse outcome are identified; such characteristics are called prognostic factors [2]. For example, previous studies suggest the risk is low among patients with venous thromboembolism (VTE) provoked by surgery, trauma, immobilization, pregnancy or female hormone intake, whereas it is higher among those with unprovoked thrombosis [3]. Stratification of patients with unprovoked VTE according to their recurrence risk might also be achieved on the basis of clinical risk factors such as gender, comorbidities or weight, or by measuring laboratory markers of thrombophilia such as factor V Leiden, the prothrombin mutation, natural coagulation inhibitor deficiencies, elevated coagulation factors and antiphospholipid antibodies [1,4-6]. More recently efforts have been made to utilise global coagulation markers, including D-dimer, as prognostic tools [1,4].

Prognostic factors have a wealth of potential uses [2]. For example, they identify groups of patients at highest risk of recurrence and thus inform prevention therapy, patient counselling and policies; they can be combined within a prognostic model to predict individual outcome risk for individuals; they allow clinicians to monitor potential changes in treatment response and outcome risk; they may reveal the causal pathway between onset and recurrence of VTE; they are potential adjustment and confounding factors in randomised trials and observational analyses; and they inform sample size and randomisation strategies in future trials [2].

Though there has been significant interest in the identification of prognostic factors for VTE recurrence, there has been less progress in the development of prognostic models. Prognostic models use multiple prognostic factors in combination to estimate outcome risk for an individual, based on their specific set of prognostic factor values [7]. Clinicians could potentially utilise the predicted risk from such models to decide when it is safe to stop therapy (often referred to as a clinical prediction rule) [1,4].

Our work aims to systematically review all the evidence for current prognostic models for VTE recurrence and adverse outcome following cessation of therapy for a first unprovoked VTE. The findings should inform clinical practice and patient care by identifying demographic, laboratory and clinical characteristics that show consistent evidence of prognostic value when adjusted for other prognostic factors, and by summarising the current prognostic models and their predictive performance. It will also inform further research of prognostic factors and models in this clinical area, including the development of a new clinical prediction rule being undertaken by the authors.

### Research aims

This systematic review will identify and summarise studies of any design examining prognostic models (and clinical decision rules based on such models) that utilise multiple prognostic factors in combination to predict the risk of VTE recurrence and/or adverse outcome in patients that have ceased therapy for a first unprovoked VTE. The patients examined must, before cessation of therapy, have received at least three months’ oral anticoagulant therapy.

## Methods/design

### Selection criteria

#### Study design

This review will include studies of any design or systematic reviews that develop, compare or validate a prognostic model (or clinical prediction rule based on a model) utilising multiple prognostic factors to predict the risk of recurrence of VTE or adverse outcome following cessation of therapy for a first unprovoked VTE.

#### Patient group

This review will study patients aged ≥18 years with a first unprovoked VTE where the patient has received at least three months’ treatment with an oral anticoagulant therapy. Studies with mixed populations (including those outside of the remit) will be included provided that the appropriate data for our defined group of patients is extractable.

#### Setting

Studies in any setting will be included.

#### Potential prognostic models

Studies must report a prognostic model utilising multiple prognostic factors to predict the risk of recurrence of VTE or adverse outcome following cessation of therapy for a first unprovoked VTE.

### Primary and secondary outcomes of our review

The primary outcome for the review will be the predictive accuracy of prognostic models, in relation to VTE recurrence. Secondary outcomes will be their predictive ability in relation to other adverse outcomes, including mortality and bleeding.

### Search strategy

The following bibliographic databases will be searched: Cochrane Library (Wiley) (to include the Cochrane Database of Systematic Reviews, DARE, HTA Databases and CENTRAL Register of Controlled Trials), MEDLINE (Ovid) 1950 to 2012, MEDLINE In - Process & Other Non-Indexed Citations (Ovid) to date and EMBASE (Ovid) 1980 to 2012. Searches will use index terms and text words that encompass the patient group supplemented by terms relating to recurrence or adverse outcome and prognostic factors (see sample MEDLINE search in Appendix 1).

Publicly available trial registers will also be searched, such as ClinicalTrials.gov, UK Clinical Research Network Study Portfolio Database (UKCRN), WHO International Clinical Trials Registry Platform and the *meta*Register of Controlled Trials (mRCT). Reference lists of all included papers will be checked and subject experts will be contacted. No restrictions on publication language or date limits will be applied.

In addition, abstracts from the following national and international conferences from 2005 onwards will be hand searched to capture studies that are not yet fully published:

•Haematology: International Society of Thrombosis and Haemostasis (ISTH), American Society of Haematology (ASH), European Haematology Association (EHA), British Society of Haematology (BSH)

•Cardiology conferences: British Cardiac Society (BCS), American College of Cardiology (ACC), European Society of Cardiology (ESC), American Heart Association (AHA), American College of Chest Physicians (ACCP)

### Study selection

This will be a two-step process. Titles (and abstracts where available) will initially be screened by two reviewers independently, using predefined screening criteria. These are broadly based on whether studies: (i) included patients with a first unprovoked VTE who received a minimum of three months’ oral anticoagulation therapy and (ii) developed or examined prognostic models in relation to VTE recurrence or other clinical outcomes. Full texts of any potentially relevant articles will then be obtained and two reviewers will independently apply the full inclusion criteria. Any discrepancies between reviewers will be resolved by discussion or by referral to a third reviewer. Portions of non-English language studies will be translated where necessary to facilitate interpretation and data extraction. The study selection process will be documented using the PRISMA flow diagram [8]. Any relevant systematic reviews identified will be screened for further primary studies. Reference management software will be used to record reviewer decisions, including reasons for exclusion.

### Data extraction

Data will be extracted independently by two reviewers using an in-depth piloted data extraction form. Disagreements will be resolved through discussion or referral to a third reviewer.

Data extraction will include the following variables:

•Study characteristics: for example, sample size, country and year

•Study design characteristics: for example, randomised controlled trial, prospective, risk of bias and length of follow-up

•Patient characteristics: for example, summaries of age, gender, family history and treatment details in the sample

•Candidate prognostic factors considered: for example, any thresholds used, methods of measurement and timing of measurement post cessation of therapy

•Outcome measures: for example, recurrence of VTE, mortality and bleeding

•Statistical methods employed and how prognostic factors included in the analysis were handled: for example, continuous or dichotomised

•Prognostic models: for example, the final model (its specification and included factors), how it was developed, and any internal or external validation performance statistics for discrimination (such as the C statistic or area under the curve) or for calibration (such as the expected/observed events ratio), together with their confidence intervals

### Assessment of study quality

The quality (risk of bias) of any studies developing or evaluating a prognostic model will be assessed using the criteria described by Altman [9], and by PROBAST (prediction study risk of bias assessment tool) if it is available before our assessments begin.

Particular elements to be considered include:

•Study design (such as whether it was a prospective design, and whether prognostic factor and outcome measurements were reliable)

•Sample size (such as whether there was a pre-specified sample size consideration accounting for numbers of events and multiple comparisons in selection of factors, and how much data was available for external validation)

•Missing data (such as adequate reporting on completeness of data, and whether imputation was used)

•Statistical analysis (such as handling of continuous variables, selection of possible factors, and use of bootstrapping or shrinkage)

•Internal and external model validation (whether model validations are reported and how these were carried out)

### Evidence synthesis

Any studies reporting a prognostic model will be summarised narratively, in particular what variables (prognostic factors) were included in the final model; how the included variables were coded; what the specification of the model was and how it produces an individual outcome probability or risk score; the reported predictive accuracy of the model; and whether the model was validated internally and/or externally, and if so how.

If multiple studies are found that validate the same prognostic model, then calibration statistics (such as expected/observed events) and discriminatory statistics (such as the C statistic or area under the curve) will be synthesised using the random-effects meta-analysis of DerSimonian and Laird [10,11], to summarise the model’s average performance across different settings and its predicted performance in a future setting [7,12].

### Economic evaluation

#### Systematic review of published cost-effectiveness studies

A systematic review will be undertaken to identify any existing economic studies including cost-effectiveness and decision model-based analyses evaluating the use of a prognostic model (or clinical prediction rule based on a model), compared to the absence of a prognostic model (or clinical prediction rule). Outcomes to be considered will include quality of life, costs and measures of cost-effectiveness (for example, incremental cost-effectiveness ratios). Studies will be identified based on the above proposed systematic review search methods with the inclusion of appropriate study design filters to identify a subset of economic studies. Quality assessment of economic evaluation studies will be performed using the criteria set out by the Consensus on Health Economics Criteria list [13], and model-based studies will be evaluated based on the guidelines described by Philips *et al*. [14].

## Discussion

It is anticipated that the results of our systematic review and meta-analysis of existing studies will be a significant step towards informing the clinical management of patients receiving therapy after a first unprovoked VTE. In particular, the results of the review will identify prognostic models for the risk of VTE recurrence or adverse outcome following cessation of therapy for a first unprovoked VTE. These will be informative for clinicians currently treating patients for a first unprovoked VTE and considering whether to stop treatment or not for particular individuals. The review will also identify areas where the evidence for or against particular candidate prognostic models is lacking, and this will lead to recommendations for initiating additional prognostic model development and validation.

The factors identified by this review as having important and consistent prognostic value will be considered for inclusion in related research by the authors (UK National Institute for Health Research HTA Project 10/94/02). This related project aims to develop a prognostic model and clinical prediction rule to identify a sub-group of patients at low risk of VTE recurrence (in whom it is considered safe to stop anticoagulation therapy given for a first unprovoked VTE) and in contrast to identify a sub-group of patients at high risk of recurrence (for whom therapy should be continued). Our systematic review will identify any current prognostic models in this area, and so – in the related project – we will compare these existing models to the performance of our own model. In the long term, if a suitable clinical prediction rule is identified, this would then lead to the comparison of groups of patients randomised to either the clinical prediction rule or usual care. Therefore, our systematic review is a crucial step towards the evidence-based use of prognostic factors and risk prediction in patients with VTE considering cessation of therapy.

## Appendix 1. Example search strategy for MEDLINE

Database: MEDLINE (Ovid) 1946 to November Week 3, 2012

Search strategy:

1. exp Venous Thromboembolism/

2. Pulmonary Embolism/

3. exp Venous Thrombosis/

4. (vte or dvt or pe).ti,ab.

5. deep vein thrombosis.ti,ab.

6. pulmonary embolism.ti,ab.

7. venous thrombo$.ti,ab.

8. or/1-7

9. (recurrence or recurr$ or re-occur$).ti,ab.

10. Recurrence/

11. exp Death/

12. (death$ or mortality).ti,ab.

13. Mortality/

14. clot$.ti,ab.

15. Hypertension, Pulmonary/

16. pulmonary hypertension.ti,ab.

17. post thrombotic syndrome.ti,ab.

18. PTS.ti,ab.

19. or/9-18

20. “Predictive Value of Tests”/

21. predict$.ti,ab.

22. exp Risk/

23. risk$.ti,ab.

24. prognos$.ti,ab.

25. or/20-24

26. exp Anticoagulants/

27. (anti-coagul$ or anticoagul$ or warfarin or acenocoumarol or coumadin or coumarin or phenprocoumon or sintrom or sinthrome or jantoven or marevan or waran or nicoumalone or dicoumarol or dicumarol).ti,ab.

28. (phenindione or dabigatran or ximelagatran or apixaban or rivaroxaban or edoxaban or azd0837 or ly517717 or ym150 or betrixaban or idraparinux).ti,ab.

29. or/26-28

30. 8 and 19 and 25 and 29

## Abbreviations

VTE: Venous thromboembolism.

## Competing interests

Dr David Moore is an associate editor for the BMC systematic reviews journal. None of the other authors have any known competing interests.

## Authors’ contributions

DF is the guarantor. JE, RR and DM drafted the manuscript. All authors contributed to the development of the selection criteria, the risk of bias assessment strategy and data extraction criteria. SB developed the search strategy. RR provided statistical expertise. DF provided expertise on venous thromboembolism. SJ contributed to the section on health economics. All authors read, provided feedback and approved the final manuscript.
